# Supplementary material for: The relationship between happiness and self-rated health: A population-based study of 19499 Iranian adults
Source: PLoS One. 2022 Mar 23;17(3):e0265914. doi: 10.1371/journal.pone.0265914 (PMC8942253; doi:10.1371/journal.pone.0265914)
Supplement: S1 Appendix — (DOCX) [file pone.0265914.s001.docx]

**S1 Table. The correlations between health and happiness in selected studies**

| **Author’s name** | **Year** | **Nation** | **Correlation** |
| --- | --- | --- | --- |
| Gaitz & Scott | 1972 | USA | r= +0.34 p< 0.01 |
| [Gehmacher](https://worlddatabaseofhappiness.eur.nl/studies/gehmacher-1987a-study-at-1987-546) | 1987 | Austria | r= +0.22 (p value was not reported) |
| [Ouweneel & Veenhoven](https://worlddatabaseofhappiness.eur.nl/studies/ouweneel-veenhoven-1994-study-zz-1980-559) | 1994 | 27 nations | r= +0.55 p< 0.01 |
| [Ventegodt](https://worlddatabaseofhappiness.eur.nl/studies/ventegodt-1995-study-dk-1993-756) | 1995 | Denmark | r= +0.28 p< 0.00 |
| [Timmermans](https://worlddatabaseofhappiness.eur.nl/studies/timmermans-1997-study-ro-1993-639) | 1997 | Romania | r= +0.24 p< 0.001 |
| [Kirkcaldy et al](https://worlddatabaseofhappiness.eur.nl/studies/kirkcaldy-et-al-2005-study-zz-1995-1621) | 2005 | 53 nations | r= +0.64 p< 0.001 |
| [Kirkcaldy et al](https://worlddatabaseofhappiness.eur.nl/studies/kirkcaldy-et-al-2005-study-zz-1995-1621) | 2005 | 53 nations | r= +0.67 p< 0.001 |
| [Landiyanto et al](https://worlddatabaseofhappiness.eur.nl/studies/landiyanto-et-al-2010-study-id-2007-15467) | 2010 | Indonesia | r= +0.03 (p value was not reported) |
| [Tienchiyava](https://worlddatabaseofhappiness.eur.nl/studies/tienchiyava-2021c-study-kz-2019-17717) | 2021 | Kazakhstan | r= +0.56 p< 0.01 |
| The current study (item 15 of OHQ and self-rated health) | 2020 | Iran | r= +0.12 p< 0.000 |

* The data are derived from world database of happiness [https://worlddatabaseofhappiness.eur.nl/search-the-database/correlational-findings/#id=-1yD-HsBSlHDfFpgD2EY].
