# Supplementary material for: The relationship between happiness and self-rated health: A population-based study of 19499 Iranian adults
Source: PLoS One. 2022 Mar 23;17(3):e0265914. doi: 10.1371/journal.pone.0265914 (PMC8942253; doi:10.1371/journal.pone.0265914)
Supplement: Attachment — Submitted filename: Response to Reviewers.docx [file pone.0265914.s003.docx]

Forough Mortazavi

Academic Editor

PLOS ONE

12 January 2022

**Dear Dr. Mortazavi,**

PONE-D-21-28962

The relationship between happiness and self-rated health: A population-based study of 19499 Iranian adults

PLOS ONE

Thank you for your e-mail. We are grateful to both reviewers and found their comments very helpful. We have revised the manuscript and now very pleased to submit the revised version for your consideration. All changed are marked in blue and hope you find it satisfactory.

Wish you all the best.

Yours sincerely

Ali Montazeri

**When submitting your revision, we need you to address these additional requirements.**

1. Please ensure that your manuscript meets PLOS ONE's style requirements, including those for file naming. The PLOS ONE style templates can be found at

<https://journals.plos.org/plosone/s/file?id=wjVg/PLOSOne_formatting_sample_main_body.pdf> and

<https://journals.plos.org/plosone/s/file?id=ba62/PLOSOne_formatting_sample_title_authors_affiliations.pdf>

Thank you. Done.

2. In your Data Availability statement, you have not specified where the minimal data set underlying the results described in your manuscript can be found. PLOS defines a study's minimal data set as the underlying data used to reach the conclusions drawn in the manuscript and any additional data required to replicate the reported study findings in their entirety. All PLOS journals require that the minimal data set be made fully available. For more information about our data policy, please see http://journals.plos.org/plosone/s/data-availability.

"Upon re-submitting your revised manuscript, please upload your study’s minimal underlying data set as either Supporting Information files or to a stable, public repository and include the relevant URLs, DOIs, or accession numbers within your revised cover letter. For a list of acceptable repositories, please see http://journals.plos.org/plosone/s/data-availability#loc-recommended-repositories. Any potentially identifying patient information must be fully anonymized.

Important: If there are ethical or legal restrictions to sharing your data publicly, please explain these restrictions in detail. Please see our guidelines for more information on what we consider unacceptable restrictions to publicly sharing data: http://journals.plos.org/plosone/s/data-availability#loc-unacceptable-data-access-restrictions. Note that it is not acceptable for the authors to be the sole named individuals responsible for ensuring data access.

We will update your Data Availability statement to reflect the information you provide in your cover letter.

This following sentences add to the manuscript.

**Data Availability**

The datasets generated for this study are fully available without restriction.

3. Please include your full ethics statement in the ‘Methods’ section of your manuscript file. In your statement, please include the full name of the IRB or ethics committee who approved or waived your study, as well as whether or not you obtained informed written or verbal consent. If consent was waived for your study, please include this information in your statement as well.

Done.

Ethics statement

The National Institutes for Medical Research Development (NIMAD), Tehran, Iran. ethics committee approved the study (IR.NIMAD.REC.l398.228). Due to the study design and all participants gave their verbal consent.

**Additional Editor Comments**

Dear authors,

Thank you for submitting your manuscript to PLOS ONE. According to the reviewers’ comments and my evaluation, several points needs careful attention.

1. The abstract should be rewritten using a structured format.

Done.

2. In the methods section, the authors should describe the sampling method and the procedures for selecting the participants in detail.

The following sentences were added to the Methods as requested:

To estimate the sample size, according to a national study [30], and based on population density, the country was classified into five categories. Then, samples were selected based on multi-stage sampling from each category. In doing so, one province was randomly selected from each category. Then two cities and two rural settings were randomly selected in each province. Every household within the city and rural areas had the same probability of being sampled. The households to be sampled were selected using systematic sampling within each census section. Finally, sampling units (the individuals) were selected randomly from all eligible persons living in the same household. Informed consent was obtained from each individual after the purpose of the study was explained. Considering the effect size of 1.4, the sample size of 20320 was estimated. However, in practice, 19499 Iranian adults were entered into the study.

3. This study was performed during the covid-19 pandemic. This may have effected on the selection of the sample.

The study was carried out just before pandemic. To satisfy the reviewer’s comment we added the following sentences to the first paragraph of Discussion:

Although 2020 coincided with the Covid-19 pandemic, and Covid-19 as a health-threatening factor can affect the level of happiness [37], we were fortunate to collect the data before the pandemic began in Iran. The first tow of deaths related to COVID-19 was reported on February 19, 2020, in Iran [38] while we collected the data in early January 2020.

4. The pandemic may have affected both the level of happiness and perceived health. This should be taken into consideration in comparisons with the findings of previous studies in the discussion section.

Please see the above.

5. PLS consider the STROBE checklist for reports of observational studies and revise the manuscript taking the following points into account:

6. Describe the setting, locations, and relevant dates, including periods of data collection.

Done.

This was a national cross-sectional study conducted from 10 January to 20 January 2020 throughout all provinces in Iran. Currently Iran has 32 provinces with over 80 million populations.

7. Describe any efforts to address potential sources of bias.

Done.

Please see strengths and limitations.

8. Explain how missing data were addressed.

Done.

Please see strengths and limitations.

9. Discuss limitations of the study, taking into account sources of potential bias or imprecision.

Done.

Please see strengths and limitations.

10. Discuss both direction and magnitude of any potential bias.

Done.

Please see strengths and limitations.

11. Discuss the generalizability of the study results.

Done.

Please see strengths and limitations.

12. Limitations must be further explained in relation to sampling method.

Thank you. There was no limitation in relation to sampling method. However, we included a separate section on strengths and limitations. Hope you find it satisfactory.

Please see strengths and limitations. Multistage sampling can simplify data collection when we have large, geographically spread samples, and we can obtain a probability sample without a complete sampling frame. However, it can lead to unrepresentative samples because large sections of populations may not be selected for sampling. Since we used multistage sampling, the result might not be generalized to all Iranians.

**Review Comments to the Author**

**Reviewer #1 Prof. Ruut Veenhoven**

This is an elementary report of a well-known relationship and is OK in itself. but adds little to knowledge. The main rationale is to show this relation in Iranian context. In the context of that goal the authors should compare the strengths of the correlations with similar studies conducted in other countries. Doing so, they might consider to compare with correlations between happiness and health gathered in the World Database of Happiness, at https://worlddatabaseofhappiness.eur.nl/search-the-database/correlational-findings/#id=-1yD-HsBSlHDfFpgD2EY. This would require that they use item 15 from the OHQ rather than the full scale.

Specifically, although the findings add a little to knowledge, the main rationale for the study could be that this relation was explored in the Iranian context. In doing so it was suggested to examine the relationship between item 15 of the Oxford Happiness Questionnaire (I am very happy) and self-rated health and see how the correlation compares with similar findings among other nations. The result showed that the correlation between item 15 and self-rated health is about 0.64 and is very similar with other studies (see S1 Appendix) [36].

The use of the OHQ should be reconsidered anyway. A look at the items shows that it covers a wide range of positive traits rather than happiness in the sense of life-satisfaction. Item 28 is about self-rated health and causes autocorrelation.

We agree. However, at this stage it is impossible to reconsider the happiness measurement. However, we added the following statement to the limitation to comply with the recommendation:

One should bear in mind that the Oxford Happiness Questionnaire covers a wide range of traits rather than happiness in the sense of life satisfaction [44]. Perhaps in future studies, if we are going to measure happiness in the sense of life satisfaction, there is a need to use an appropriate measure. In addition, item 28 of the Oxford Happiness Questionnaire is about self-rated health, and thus it might cause autocorrelation with the self-rated health measure, although the item 28 and the measure of self-rated health are worded differently. The latter is negative (I do not feel particularly healthy) while the former is positive and askes people to rate their current health.

In the discussion section the authors acknowledge that the correlation may be driven by an effect of happiness on health, but in the conclusion they attribute the correlation to the effects of health on happiness.

Thank you for your comment. We agree that we made a mistake and thus the paragraph was deleted.

**Reviewer #2: Dr. Vahid Rashedi**

The results of this paper are interesting, and can contribute to the literature on happiness and health among adults. However, several revisions are required for this work to be accurately portrayed, received, and interpreted. Below, I have separated my comments into an overall comment.

Thank you for your positive evaluation and comments.

- The first paragraph should include some comparative statistics on figures/statistics from EMRO region countries to provide the context for Iran being similar (in terms of happiness and health).

According to the world happiness report (2017-2019), the highest and lowest happiness scores were for Finland and Afghanistan, respectively. The Islamic Republic of Iran ranked 118th among 153countries. Although happiness score in Iran was lower than some countries in the Eastern Mediterranean Region (EMRO), such as Saudi Arabia, Pakistan, Morocco, but was higher than some other countries including Jordan, Tunisia, and Egypt [6].

One of the most frequently used measures of self-reported health status is a single question asking individuals to rate their overall health on a scale from excellent to very poor. There is widespread agreement that this simple global question provides a useful summary of how individuals perceive their overall health status [7]. The results of a cross-national study that compared health in Egypt, Iran, Jordan, and the United States showed that means and standard deviations of self-rated health by country was (2.79±0.85), (2.99±0.81), (3.06±0.83), and (3.23±0.78), respectively [8].

- The validity and reliability of the questionnaire (Oxford standard Happiness Questionnaire & Self-rated health) among the Iranian population should be justified in detail.

Thank you. Both of them were added in detail.

Psychometric properties of the Iranian version of questionnaire are well documented. Cronbach's alpha coefficient (measure of internal consistency) and interclass correlation coefficient (measure of stability) were 0.90 and 0.79, respectively. The convergent and divergent validity of the questionnaire were high and acceptable [32].

Validity and reliability of self-rated health measure among Iranian showed acceptable results. The criterion validity showed that the self-rated health and the WHO-5 well-being had positive correlation as expected (r= 0.5, p< 0.001). Additionally, the reliability of the self-rated health, using interclass correlation coefficient (ICC), was found to be 0.83; 95% CI (0.72 to 0.90) [35].

- In table 1; write the scale of Education & Income.

The scales were included.

Education: year

Income: self-reported

- More discussion about the justification of finding should be provided in this section. The discussion is disorganized and confusing. It is better to summarize your findings firstly and then discuss them separately.

Done.

Please see discussion

- The conclusion is the repetition of what has been said in the text! And need a revision based on results.

Thank you for your comment. The conclusion was revised.

It seems that adopting policies to improve public health and placing health on the public agenda could be an effective approach for increasing happiness.

- The manuscript needs a revision for grammar, typos, and English expressions to improve the readability.

Thank you. The manuscript was copy edited once more.
