# Supplementary material for: The relationship between happiness and self-rated health: A population-based study of 19499 Iranian adults
Source: PLoS One. 2022 Mar 23;17(3):e0265914. doi: 10.1371/journal.pone.0265914 (PMC8942253; doi:10.1371/journal.pone.0265914)
Supplement: Attachment — Submitted filename: Response to Reviewers.2.docx [file pone.0265914.s004.docx]

Forough Mortazavi

Academic Editor

PLOS ONE

1 February 2022

**Dear Dr. Mortazavi,**

PONE-D-21-28962

The relationship between happiness and self-rated health: A population-based study of 19499 Iranian adults

PLOS ONE

Thank you for your e-mail and the comments. We have revised the manuscript and now pleased to submit the second revision for your consideration. Hope you find revisions satisfactory.

Wish you all the best.

Yours sincerely

Ali Montazeri

**Editor Comments:**

Please kindly consider the points raised by reviewer 1 regarding item 28 and auto-correlation.

Thank you for your comment. The point was responded. Please see reviwer1.

In presenting the results of statistical analyses, please describe how the authors divided the sample into two groups of low and high happiness for logistic regression analyses. This section is in need of further clarification.

The following sentence was added to the statistical analysis as requested:

Logistic regression analyses were performed to assess the relationship between happiness and independent variables, including participants’ health status. As such happiness as dependent variables was categorized into: ‘happy’ (scores ranging from 4 to 6) and ‘unhappy’ (scores ranging from 1 to 3).

Also, in the titles of tables 2 and 3, PLS replace 'happiness' with the correct description, i.e. 'Oxford happiness scores’.

Thank you. Done.

**Reviewers'Comments to the Author**

**Reviewer #1 Prof. Ruut Veenhoven**

My objection about auto-correlation is not met. The answer that the concerned questions on health were formulated differently does not convince. Without re-analysis of the data I cannot recommend acceptance.

Thank you for your comment. The correlation between happiness without item 28 and self-reported health examined and now was added to Appendix.

Perhaps in future studies, if we are going to measure happiness in the sense of life satisfaction, there is a need to use an appropriate measure. In addition, item 28 of the Oxford Happiness Questionnaire is about self-rated health, and thus it might cause autocorrelation with the self-rated health measure, although the item 28 and the measure of self-rated health are worded differently. The latter is negative (I do not feel particularly healthy) while the former is positive and askes people to rate their current health. However, we did examine this correlation and found the correlation coefficient to be 0.17 (See S1 Appendix).

**Reviewer #2: Dr. Vahid Rashedi**

I read the manuscript with great interest and think the data reported in this study is valuable, and the authors have made all the corrections to the article entitled: "The relationship between happiness and self-rated health: A population-based study of 19499 Iranian adults".

Thank you for comments and time spent to review this paper.
