# Supplementary material for: The relationship between happiness and self-rated health: A population-based study of 19499 Iranian adults
Source: PLoS One. 2022 Mar 23;17(3):e0265914. doi: 10.1371/journal.pone.0265914 (PMC8942253; doi:10.1371/journal.pone.0265914)
Supplement: Attachment — Submitted filename: Response to Reviewers.3.docx [file pone.0265914.s005.docx]

Forough Mortazavi

Academic Editor

PLOS ONE

23 February 2022

**Dear Dr. Mortazavi,**

PONE-D-21-28962

The relationship between happiness and self-rated health: A population-based study of 19499 Iranian adults

PLOS ONE

Thank you for your e-mail and the comments. We have revised the manuscript and now pleased to submit the third revision for your consideration. Hope you find revisions satisfactory.

Wish you all the best.

Yours sincerely

Ali Montazeri

**Editor Comments:**

Based on comments by reviewer #1 and my own evaluation, an important point raised still remains unaddressed. PLS take note that without the approval of reviewer # 1, the manuscript cannot be accepted for publication. I strongly recommend that you reanalyze the data after removing the item pointed out by reviewer #1.

Thank you. All comments were attended as suggested. The following revisions were applied:

1. Methods (Statistical analysis):

Data were explored using descriptive statistics, including frequency, percentage, mean and standard deviation. Logistic regression analyses were performed to assess the relationship between happiness and independent variables, including participants’ health status. However, since some eminent scholars [36] believe that there is an auto-correlation between item 28 and the self-rated health, we did reanalyze the data while item 28 (I do not feel particularly healthy) was excluded from the Oxford happiness score. As such for both with and without item 28 of the Oxford questionnaire, happiness as dependent variables were categorized into: ‘happy’ (scores ranging from 4 to 6) and ‘unhappy’ (scores ranging from 1 to 3). The results expressed as odds ratio and 95% confidence intervals. A significant level was set at P< 0.05.

2. Results:

The results are shown in Table 3. In addition, the results obtained from the same analysis when items 28 was excluded are shown in Table 4. The results almost were very similar and no significant difference was observed from the previous analysis except for age 18-24 (OR:1.191, 95% CI, P= 1.038-1.367, p= 0.013) and 6-9 years of education (OR:1.118, 95% CI, P= 1.024-1.221, p= 0.013).

3. Strengths and limitations

In addition, item 28 of the Oxford Happiness Questionnaire is about self-rated health, and thus it might cause autocorrelation with the self-rated health measure, although the item 28 and the measure of self-rated health are worded differently. The latter is negative (I do not feel particularly healthy) while the former is positive and askes people to rate their current health. However, as indicated in reanalysis of the data (Table 4), the findings did not show any major differences to our earlier analysis as shown in Table 3.
