# Supplementary material for: The relationship between happiness and self-rated health: A population-based study of 19499 Iranian adults
Source: PLoS One. 2022 Mar 23;17(3):e0265914. doi: 10.1371/journal.pone.0265914 (PMC8942253; doi:10.1371/journal.pone.0265914)
Supplement: Attachment — Submitted filename: Response to Reviewers.R4.docx [file pone.0265914.s006.docx]

Forough Mortazavi

Academic Editor

PLOS ONE

8 March 2022

**Dear Dr. Mortazavi,**

PONE-D-21-28962

The relationship between happiness and self-rated health: A population-based study of 19499 Iranian adults

PLOS ONE

Thank you for your e-mail and the comments. We have revised the manuscript and now pleased to submit the fourth revision for your consideration. Hope you find revisions satisfactory.

Wish you all the best.

Yours sincerely

Ali Montazeri

**Review Comments to the Author**

**Reviewer #1 Prof. Ruut Veenhoven**

In an earlier comment I noted that the Oxford happiness scale involves an item on health, which causes auto correlation. I advised to recalculate, leaving this item out.

Thank you. The result now is presented Table2.

I also noted that this questionnaire covers broader matters than happiness as discussed in the introduction, and advised to calculate correlations for the few items on life-satisfaction separately The authors did not recalculate, but sufficed mentioning these points under limitations. In my view, that is not acceptable.

Thank you. Done.

**Method**

Since this questionnaire covers broader matters than happiness [33], we calculated correlations for the few items on life satisfaction with self-rated health separately.

**Result**

In addition, the results obtained from correlation between few items on life satisfaction and self-rated health in are presented in Table3. Overall the findings showed a relatively low correlation (r= 0.15).

**Table3**

Still another point: the authors write Despite the importance of studying the relationship between happiness and health, only a few 81 small-scale studies have been conducted in Iran [21- 24] They are apparently unaware of the 337 findings on this topic listed in the World Database of happiness at <https://worlddatabaseofhappiness.eur.nl/search-the-database/correlational> findings/#id=N1iFWn8BRfHHvZHVJh7X

Thank you for your comment. We agree that we made a mistake, and thus the paragraph was edited.

Although there are several many Iranian studies on happiness [21], it seems that the relationship between happiness and health is overlooked.
